# Surgical Assessment of Tissue Quality during Pelvic Organ Prolapse Repair in Postmenopausal Women Pre-Treated Either with Locally Applied Estrogen or Placebo: Results of a Double-Masked, Placebo-Controlled, Multicenter Trial

**DOI:** 10.3390/jcm10112531

**Published:** 2021-06-07

**Authors:** Marie-Louise Marschalek, Klaus Bodner, Oliver Kimberger, Raffaela Morgenbesser, Wolf Dietrich, Christian Obruca, Heinrich Husslein, Wolfgang Umek, Heinz Kölbl, Barbara Bodner-Adler

**Affiliations:** 1 Department of General Gynecology and Gynecologic Oncology, Medical University Vienna, 1090 Vienna, Austria; marie-louise.marschalek@meduniwien.ac.at (M.-L.M.); klausbodner@yahoo.com (K.B.); raffaela.morgenbesser@meduniwien.ac.at (R.M.); heinrich.husslein@meduniwien.ac.at (H.H.); wolfgang.umek@meduniwien.ac.at (W.U.); heinz.koelbl@meduniwien.ac.at (H.K.); 2 Department of Anesthesiology, Medical University of Vienna, 1090 Vienna, Austria; oliver.kimberger@meduniwien.ac.at; 3 Department of Gynecology and Obstetrics, University Hospital Tulln, 3430 Tull, Austria; Wolf.Dietrich@kl.ac.at (W.D.); gyn-geburtshilfe@tulln.lknoe.at (C.O.); 4 Karl Landsteiner Institute, Department of Special Gynecology and Obstetrics, 3100 St. Pölten, Austria

**Keywords:** pelvic organ prolapse, local estrogen therapy, postmenopausal women, surgical outcome

## Abstract

The aim of this prospective randomized, double-masked, placebo-controlled, multicenter study was to analyze the surgeon’s individual assessment of tissue quality during pelvic floor surgery in postmenopausal women pre-treated with local estrogen therapy (LET) or placebo cream. Secondary outcomes included intraoperative and early postoperative course of the two study groups. Surgeons, blinded to patient’s preoperative treatment, completed an 8-item questionnaire after each prolapse surgery to assess tissue quality as well as surgical conditions. Our hypothesis was that there is no significant difference in individual surgical assessment of tissue quality between local estrogen or placebo pre-treatment. Multivariate logistic regression analysis was performed to identify independent risk factors for intra- or early postoperative complications. Out of 120 randomized women, 103 (86%) remained for final analysis. Surgeons assessed the tissue quality similarity in cases with or without LET, representing no statistically significant differences concerning tissue perfusion, tissue atrophy, tissue consistency, difficulty of dissection and regular pelvic anatomy. Regarding pre-treatment, the rating of the surgeon correlated significantly with LET (r = 0.043), meaning a correct assumption of the surgeon. Operative time, intraoperative blood loss, occurrence of intraoperative complications, total length of stay, frequent use of analgesics and rate of readmission did not significantly differ between LET and placebo pre-treatment. The rate of defined postoperative complications and use of antibiotics was significantly more frequent in patients without LET (*p* = 0.045 and *p* = 0.003). Tissue quality was similarly assessed in cases with or without local estrogen pre-treatment, but it seems that LET prior to prolapse surgery may improve vaginal health as well as tissue-healing processes, protecting these patients from early postoperative complications.

## 1. Introduction

Since estradiol receptors α and β (ESR1/2) were found in the squamous epithelium of the bladder, urethra, vagina and anal canal [1] as well as in the paraurethral tissues such as urethral sphincter, uterosacral ligaments and pelvic floor muscles [2], it is clear that the pelvic organs and their surrounding muscular and connective tissue support are estrogen-responsive.

For some time, local estrogen therapy (LET) has become the focus of interest in the treatment of pelvic floor disorders. There is some evidence that local estrogens might improve the health of the vaginal epithelial layer [3,4], but it is yet unclear if hormones are able to improve the connective tissue support [5]. Little research with different results has been carried out on determining the effect of preoperative locally applied estrogen on the tissue quality as well as on the intraoperative and early postoperative course in postmenopausal patients undergoing surgical prolapse repair. In one study, evaluating the role of locally applied estrogen prior to POP surgery, no statistically significant increase in the thickness of the vagina in the treatment group compared to the placebo group could be observed [6]. Otherwise, pre-treatment with vaginally applied estradiol has shown easier tissue handling and significant cervical ripening in postmenopausal women prior to operative hysteroscopy [7]. Furthermore, an RCT with preoperative LET reported decreased matrix metalloprotease activity but increased collagen content and vaginal wall thickness. The authors concluded that this might be an improvement of the substrate for suture placement at the time of surgical repair [5]. 

Based on these findings, preoperative local estrogen treatment may also facilitate surgical prolapse repairs by improving tissue quality and texture. Moreover, if complications during surgery could be minimalized, the intraoperative and early postoperative outcome might also be improved. 

The aim of this trial was to analyze the surgeon’s subjective intraoperative assessment of tissue quality during pelvic floor surgery. Our study hypothesis is that there is no significant difference in surgical assessment of tissue quality between pre-treatment with or without local estrogen. Additionally, we determined the intraoperative as well as the early postoperative course in postmenopausal cases undergoing prolapse surgery with or without local estrogen pre-treatment. The main variables of interest included conditions during surgery determined by the surgeon blinded to treatment allocation and assessed by an 8-item questionnaire. 

## 2. Materials and Methods

### 2.1. Study Design

This was a prospective randomized, double-blind, placebo-controlled multicenter study conducted at the department of General gynecology and gynecologic oncology of the Medical University of Vienna (MUVI) (main study center), as well as at the department of obstetrics and gynecology of the University Hospital of Tulln (second center), Austria. The study was approved by the institutional review board at both centers (IRB number: 1706/2016), and each subject provided written informed consent to participate. All eligible patients were referred by a gynecologist and seen by a urogynecologist at the respective center. The trial was registered in ClinicalTrials.gov (NCT03779633, https://clinicaltrials.gov/ct2/show/NCT03779633, accessed on 1 June 2021) and EudraCT (Nr. 2016-000410-30).

### 2.2. Patient Selection

Postmenopausal women with symptomatic POP and planned surgical prolapse repair were eligible participants. In addition, eligible participants needed to be capable of applying a vaginal cream and comprehend the informed consent as well as the delivered questionnaire. Patients with a suspicion or history of malignancies, postmenopausal bleeding, a history of deep vein thrombosis, inherited or acquired blood clotting disorders, a history of transient ischemic attack, myocardial infarction or ischemic heart disease, hypersensitivity or contraindications to estrogen and language barrier were excluded.

### 2.3. Intervention and Study Procedures

Women were randomly assigned 1:1 to estradiol containing Linoladiol (Montavit company, Absam, Austria) cream (intervention group) or placebo cream (control group). The active ingredient of Linoladiol Estradiol-Emulsion is 0.10 mg estradiol in 1 g cream and is chemically and biologically identical to endogenous human estradiol. Placebo cream contained cetyl alcohol, probylene glycol, triglycerides, hostacerin T3, polysorbate, almond oil, benzyl alcohol and purified water. The study medication was provided by the Pharmaceutical Company Montavit Ges.m.b.H. (Absam, Austria).

Women were instructed to use the cream (estrogen as well as placebo) intravaginally once daily for one week, every 48 h for the following week, and then twice weekly for the remaining 4 weeks (total treatment duration 6 weeks). Participants documented the self-administered application in a patient diary. Adherence was assessed at the follow-up visit after six weeks by the trial coordinators. 

### 2.4. Randomization

Patients who had consented to participate and met the eligibility criteria were randomly allocated to either receive estradiol-containing cream or placebo cream in a 1:1 ratio. The randomization was conducted by the Pharmaceutical Company Montavit. The allocation sequence was computer-generated by Rancode Professional 3.6 software (IDV, Gauting, Germany) A randomization in blocks of four was performed and carried out from numbers 1 to 400. On the basis of the randomization list, all labels were produced, and information of the principal investigator as well as the randomization number was included. The research team was unaware of each participant’s allocated treatment group. The only unblinded person was the study coordinator of the company Montavit Ges.m.b.H.

### 2.5. Measurements

The primary efficacy outcome of this study was the surgeon’s individual assessment of tissue quality during pelvic floor surgery, assessed by an 8-item questionnaire. Secondary outcomes included intraoperative and early postoperative course of the two groups. 

#### 2.5.1. Intraoperative Surgical Assessment of Tissue Quality in Cases with or without Estrogen Pretreatment

All surgical interventions were carried out by 3 senior consultants who are trained and specialized in pelvic floor surgery. To provide an objective assessment, the surgeons were blinded to the pretreatment of the patient. As there is no validated questionnaire available for assessing tissue quality during pelvic floor surgery, the questionnaire was self-developed by the authors in German language. After the surgical procedure was completed, the responsible surgeon filled out an 8-item questionnaire. The questionnaire was completed immediately following the operative procedure to minimize recall bias. The questions referred to the surgeon’s subjective appraisal of tissue characteristics and conditions during the surgery. 

A three- to four-point scoring system was used in four questions to assess the degree of tissue quality and surgical difficulty. Furthermore, four questions requested tissue dissection and pelvic anatomy and could be answered with a “yes” or “no” response. In detail, the following surgical conditions were evaluated:

- perfusion of the tissue within the operation area (four-point scoring system);
- atrophy with poor perfusion within the operation area (four-point scoring system);
- presence of a soft or firm tissue consistency (three-point scoring system);
- difficulty of dissection of delineating surgical planes (four-point scoring system);
- tissue handling (yes/no);
- regular pelvic anatomy (yes/no).

**Figure 1:**
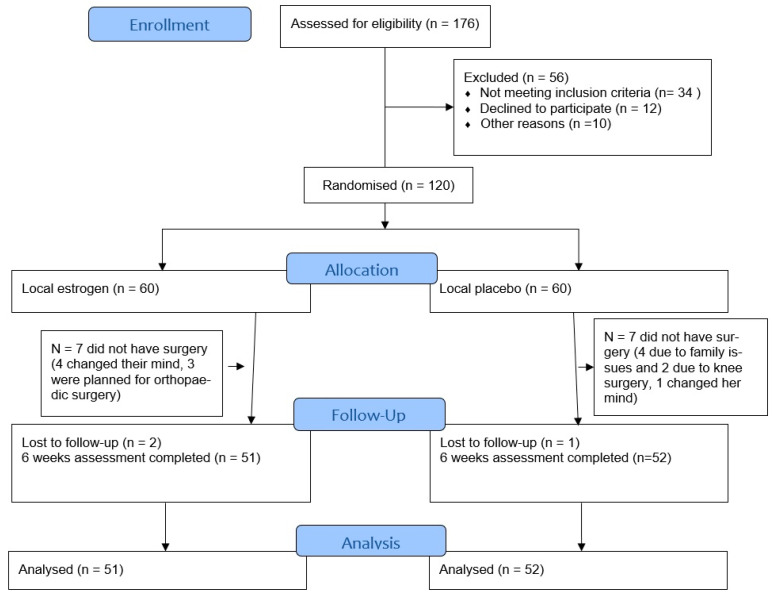
Flow of participants through the trial.

Finally, surgeons were questioned whether they thought the patient had received pre-treatment with local estrogen cream or not (yes/no answer). The detailed questionnaire is shown in Figure 1.

#### 2.5.2. Intraoperative and Early Postoperative Surgical Course in Cases with or without Estrogen Pre-Treatment

Intra- and early postoperative courses were compared between the two study groups. The following parameters were documented: type of pelvic floor surgery including uterus preservation or prolapse hysterectomy, affected vaginal compartment (anterior, apical or posterior), operative time (time interval between the skin (mucosal) incision and the completion of the skin closure in minutes), occurrence of intraoperative complications (in detail: injury of urinary bladder, ureter, bowel, or vessels, ureter kinking), and significant intraoperative blood loss (estimated blood loss > 500 mL).

For early postoperative course, the following parameters were documented during the patient’s hospital stay: length of stay (days), frequent postoperative use of analgesics (administration of intravenous analgesics more than 2 times per day), postoperative use of antibiotics and defined postoperative complications (complications associated with the pelvic floor surgery). Defined complications included in detail postoperative urinary tract infections (UTIs), postoperative urinary retention (POUR), surgical site infection, secondary bleeding and readmission (admission within 30 days of discharge).

### 2.6. Statistical Analysis

Chi-square was used for the comparison of categorical variables between the two groups (intervention versus placebo group) and Student’s t-test for continuous variables. The average score of the first four questions in the questionnaire was reported as mean and standard deviation (SD). For correlation analysis the Spearman test was used with correlation coefficient. A generalized linear model was performed to identify independent parameters associated with complications related to pelvic floor surgery. Our null hypothesis was that there was no significant difference in individual surgical assessment of tissue quality between postmenopausal women pre-treated with LET or placebo cream. To discard this null hypothesis, the sample size was calculated using preliminary, unpublished data from preliminary tests of the surgical questionnaire as follows: The G-Power 3.1.9.2 software (Universität Düsseldorf, Düsseldorf, Germany) was used for calculation of sample size using Student’s t test for unpaired groups with a level of significance at 5%, a test power of 80% and an effect size of 0.45. Thus, 60 patients per group were calculated. A *p* value < 0.05 was considered statistically significant. The SPSS system (IBM, Armonk, NY, USA, Version 23) was used for the calculations.

## 3. Results

One hundred twenty women were randomized between 2017 and 2020 to receive either vaginal estrogen cream (*n* = 60) or placebo cream (*n* = 60) 6 weeks before their planned prolapse surgery. Recruitment took place in two urogynecology centers in east Austria (Medical University of Vienna as the main study center and University Hospital Tulln). Seventeen patients in all were either lost to follow-up between recruitment and planned surgery or they postponed/cancelled their surgical appointment. Finally, 103/120 (86%) women were available for primary data analysis (Figure 1). 

The mean age of our study cohort was 62.8 (±10.0) years. Majority of the baseline characteristics were comparable between the two groups (*p* > 0.05), except for uterus preserving surgery, which was significantly more frequently performed within the placebo group (34.6% vs. 21.6%; *p* = 0.043). Ninety-six (93.2%) surgeries were performed by vaginal route, and 7 (6.8%) cases were operated by a laparoscopic route. Uterus preservation with sacrospinous hysteropexy +/− anterior/posterior colporrhaphy was performed in 29 cases, and 55 women underwent vaginal prolapse hysterectomy with Mc Call culdoplasty +/− anterior/posterior colporrhaphy. Nineteen women presented with vaginal vault prolapse as they had a history of previous hysterectomy. The majority of these cases received a reconstructive pelvic floor surgery, and only in one case was an obliterative procedure with Neugebauer Le-Fort colpocleisis performed. In this particular case a dissection of vesicouterine and rectovaginal space was not appropriate; therefore, the assessment “easy peel of the anterior and posterior vaginal mucosa” was made. Regarding urinary incontinence, a two-stage procedure is recommended at our institution. None of our patients underwent urinary incontinence treatment at the same time of prolapse surgery. None of the included patients had received hormone replacement therapy (HRT).

Baseline characteristics of the two study groups are presented in Table 1.

### 3.1. Safety

No serious adverse events were recorded in the estrogen or placebo groups.

### 3.2. Intraoperative Surgical Assessment of Tissue Quality

During pelvic floor surgery, surgeons assessed the tissue quality similarity in cases with or without estrogen pre-treatment, representing no statistically significant differences concerning tissue perfusion, tissue atrophy, tissue consistency, difficulty of dissection and regular pelvic anatomy (Table 2).

When questioned whether surgeons assume that a patient had received pre-treatment with local estrogen or not, the rating of the surgeon correlated significantly with estrogen treatment (*r* = 0.043), meaning a correct assumption of the surgeon concerning pretreatment. 

### 3.3. Intra- and Early Postoperative Course in Patients with or without Estrogen Pre-Treatment

Operative time, intraoperative blood loss, occurrence of intraoperative complications as well as total length of stay, frequent use of analgesics and rate of readmission did not significantly differ between estrogen and placebo pre-treatment (*p* > 0.05). 

However, the rate of defined postoperative complications could be detected significantly more frequently in the placebo group compared to the intervention group (*p* = 0.045). In detail, UTI, postoperative hemorrhage as well as surgical site infection occurred more frequently in cases without estrogen pre-treatment (*p* = 0.045).

Similarly, use of antibiotics was significantly more frequent in women who received placebo compared to women who received estrogen (*p* = 0.003). 

### 3.4. Multivariate Analysis

Multivariate logistic regression analysis revealed that age, estrogen treatment, BMI, affected vaginal compartment, smoking, COPD, and POP-Q at baseline could not be identified as independent risk factors for intra- or postoperative complications (Table 3).

## 4. Discussion

This randomized, placebo-controlled, double-blind, multicenter trial investigated the effects of locally applied estrogen pre-treatment on tissue quality and handling during prolapse surgery (subjective surgical assessment). Furthermore, intra- and early postoperative surgical course was also assessed in both study groups.

### 4.1. Main Results

Our results showed that surgeons similarly assessed the tissue quality during prolapse surgery in cases with or without estrogen pre-treatment, but interestingly the rating of the surgeon correlated significantly with estrogen pre-treatment, meaning a correct assumption of the surgeon concerning the estrogen pre-treatment arm. Furthermore, we found significantly more urinary tract infections, postoperative hemorrhage as well as surgical site infections in cases without estrogen pre-treatment, and the use of antibiotics was significantly more frequent in women who received placebo. 

### 4.2. Comparison with Literature

As surgery for POP currently has a lifetime risk of 12–19% and is expected to increase in the future [8,9], it is of utmost importance to seek possibilities to reduce perioperative complications of POP surgery.

Reconstructive pelvic floor surgery usually includes dissection of connective tissue planes, whereas adherent planes could make dissection during surgery more difficult. On the other hand, difficulties in dissection or tissue handling increase the incidence of intraoperative complications such as bladder/visceral injury, bleeding and so on [10,11]. In particular, in cases with post-hysterectomy prolapse, the vaginal wall tends to be thin; thus, vaginal dissection becomes difficult, and unintended opening of the peritoneum may occur [12].

Therefore, it is important to investigate agents that are able to improve surgical outcomes, reduce risks of difficult dissection and allow a safe and effective surgical procedure.

In postmenopausal women, vaginal atrophy, and consequently thinning of the vaginal wall, can make surgical dissection more difficult during anterior and posterior vaginal wall repairs. In our study, surgeons did not find an advantage of preoperative estrogen concerning dissection. 

To our best knowledge, this is the first study evaluating surgeon’s assessment of tissue quality in prolapse cases with estrogen pre-treatment. Although surgeons did not notice any significant differences regarding tissue quality in detail, a correct assumption concerning the estrogen pre-treatment arm was observed. The authors may hypothesize that this finding might be explained by the design of the questionnaire. However, this has no impact on the patient’s clinical outcome or postoperative course. Only a few studies investigating the structure of tissue are available in the literature, in which mainly objective and not subjective tissue markers were determined. Tyagi et al., for example, demonstrated an increase in structural proteins, collagen and elastin in women with severe POP after preoperative LET [13]. Another RCT with preoperative LET reported decreased matrix metalloprotease activity but increased collagen content and vaginal wall thickness up to the time of prolapse surgery. The authors concluded that this might be an improvement of the substrate for suture placement at the time of surgical repair [5]. Yet, so far there is no evidence to suggest that a strengthened vaginal tissue after local estrogen reduces vaginal tearing and bleeding or promotes tissue healing. Further, we could not find any studies that focused on subjective assessment of tissue quality improvement and estrogen pre-treatment in prolapse patients. 

Moreover, our results demonstrated some benefits for patients with LET prior to surgery, as these cases showed fewer UTIs, hemorrhage and surgical site infections during their postoperative course. One may hypothesize that local estrogen may improve vaginal health and environment, as well as tissue healing processes, protecting these patients from early postoperative complications.

In general, postmenopausal women may be at higher risk of surgical site infections due to changes in the vaginal flora. Urinary tract infection is the most common complication in prolapse surgery with an incidence ranging from 9% to 31% [14,15,16]. It is known that local estrogen treatment improves genitourinary syndrome in menopause, which includes urinary symptoms of urgency or recurrent urinary tract infection [17,18]. As estrogens are vasoactive hormones, they are able to increase blood flow, which helps to maintain the low pH in the vagina necessary to protect the patient from UTIs and vaginitis [19]. 

Concerning the role of perioperative estrogen on urinary tract infections, there is less evidence. Preoperative estrogens unexpectedly reduced the incidence of postoperative urinary tract infections in an RCT evaluating estrogen before vaginal operations for genital prolapse [20]. A systematic review, published in 2015, reported that preoperative vaginal estrogen decreased the frequency of bacteriuria in the first postoperative month after prolapse repair [21]. Further, a randomized controlled trial (RCT), conducted by Karp et al., investigated the impact of an estradiol-releasing ring 2 weeks after pelvic floor repair and found improved markers of tissue quality and slightly reduced urinary tract infections compared to placebo medication [22]. This is in line with our findings.

### 4.3. Strengths and Limitations

Strengths of our study include the study design and the large study population with excellent participant retention, medication compliance and low drop-out rates. Furthermore, as surgeons were blinded to preoperative treatment, bias of preconceived beliefs could significantly be diminished. As the surgical interventions were carried out by three different surgeons, the authors accept that interpersonal bias could have occurred; therefore, the assessment by one surgeon may have been preferable. In addition, the questionnaire, although elaborated by experienced surgeons, is not a validated and often-used questionnaire. However, to date, there is no validated questionnaire for assessing tissue quality. Furthermore, the duration of 6 weeks’ preoperative local estrogen treatment might be too short to show a representative effect on the tissue or outcome. Additionally, the missing baseline assessment of tissue quality may be defined as a limitation factor and results in a restriction of outcome interpretation. Although our patients represent a relatively homogenous surgical pool (involving over 90% vaginal prolapse procedures with native tissue repair), different surgical interventions for POP exist. As a result, depending on the type of surgery, different outcomes can occur due to the different surgical treatment modalities. This fact clearly has to be mentioned as a limitation factor.

## 5. Conclusions

We observed that surgeons did not notice any significant differences regarding tissue quality during prolapse surgery between patients with or without estrogen pre-treatment. However, local estrogen might improve vaginal health, as well as tissue-healing processes, protecting these patients from early postoperative complications. This is because fewer UTIs, hemorrhage and surgical site infections could be observed in the estrogen pre-treatment group. Our findings are of clinical relevance as POP and POP surgery is expected to increase in the future; therefore, it is of most importance to seek possibilities to reduce perioperative complications during prolapse surgery.

## Figures and Tables

**Table 1:** Baseline characteristics of the study population (*n* = 103).

Characteristic	Estrogen Group (*n* = 51)	Placebo Group (*n* = 52)	*p*-Value
	Mean ± SD or *n* (%)	Mean ± SD or *n* (%)	
Age (y)	64.3 ± 9.7	61.2 ± 10.1	n.s.
Age at menopause (y)	48.9 ± 6.2	49.5 ± 5.9	n.s.
Parity	2.4 ± 2.1	2.3 ± 1.1	n.s.
BMI (kg/m^2^)	26.9 ± 4.0	27.3 ± 4.8	n.s.
Diabetes	4 (8)	4 (8)	n.s.
COPD	4 (8)	3 (6)	n.s.
Smoking	11 (22)	8 (15)	n.s.
POP-Q stage baseline & after 6 weeks	2.8 ± 0.4	2.7 ± 0.5	n.s.
Stage II	9 (17)	13 (25)	
Stage III	41 (80)	38 (73)	
Stage IV	1 (0)	1 (0)	
Uterine Preservation			0.043
No	26 (51)	29 (56)	
Yes	11 (22)	18 (35)	
Previous hysterectomy	14 (28)	5 (10)	
Affected vaginal compartment			n.s.
Anterior (exclusive)	3 (6)	4 (8)	
Posterior (exclusive)	1 (2)	1 (2)	
all three (anterior, apical, posterior)	47 (92)	47 (90)	
Operative time	88.73 ± 30.3	78.46 ± 32.2	n.s.
Total length of stay	4.5 ± 1.2	4.6 ± 1.2	n.s.
Significant blood loss (>500 mL)	1 (2)	1 (2)	n.s.
Use of analgesics	51 (100)	52 (100)	n.s.
Use of antibiotics	3 (6)	15 (29)	0.003
Readmission	3 (6)	2 (4)	n.s
Defined complication	11(22)	15(29)	0.045
POUR	6/11 (55)	4/15 (27)	
UTI	3/11 (27)	6 (40)	
Postoperative hemorrhage	1/11(9)	2 (13)	
surgical site infection	1/11 (9)	3 (20)	

BMI, body mass index; COPD, chronic obstructive pulmonary disease; POP-Q, Pelvic Organ Prolapse Quantification System; POUR, postoperative urinary retention; UTI, urinary tract infection; Data are mean ± SD or *n* (%); n.s. = not significant *p* > 0.05.

**Table 2:** Intraoperative subjective surgical assessment of the tissue quality in patients with estrogen or placebo pre-treatment.

	Estrogen Group (*n* = 51) Mean ± SD or *n* (%)	Placebo Group (*n* = 52) Mean ± SD or *n* (%)	*p*-Value
**Q1. The tissue within the operation area is well perfused.**	2.12 ±1.03	2.19 ± 0.99	0.219
**Q2. There is atrophy and poorly perfused tissue within the operation area.**	0.55 ± 0.95	0.54 ± 0.96	0.836
**Q3. The tissue consistency within the operation is (soft/firm)**	0.16 ± 0.42	0.15 ± 0.36	0.435
**Q4. The surgical planes are easily dissected of delineated.**	1.67 ± 1.16	1.88 ± 1.15	0.225
**Q5. Easy vesicovaginal dissection**			0.518
**1: no**	8 (16)	9 (17)	
**2: yes**	43 (84)	43 (82)	
**Q6. Easy rectovaginal dissection**			0.486
**1: no**	9 (18)	10 (19)	
**2: yes**	42 (82)	41 (79)	
**3: Pouch of Douglas was not opened**	0 (0)	1 (2)	
**Q7. The pelvic anatomy is regular**			0.597
**1: no**	7 (14)	7 (14)	
**2: yes**	44 (86)	45 (87)	
**Q8. Do you think the patient received pretreatment with local estrogen cream?**			0.034
**1: no**	12 (24)	22 (42)	
**2: yes**	39 (77)	30 (58)	

Data are mean ± SD or *n* (%).

**Table 3:** Multiple logistic regression analysis to identify independent risk factors for complications with the covariates age, estrogen pre-treatment, BMI, affected compartment, smoking, COPD and POP-Q at baseline.

Variable	OR (95% CI)	*p*-Value
Age	0.993 (0.945–1.043)	0.780
Estrogen treatment	0.475 (0.177–1.274)	0.139
BMI	0.984 (0.885–1.093)	0.758
Affected vaginal compartment	1.338 (0.636–2.815)	0.443
Smoking	1.223 (0.331–4.522)	0.763
COPD	1.624 (0.143–18.485)	0.696
POP-Q at baseline	0.454 (0.147–1.400)	0.169

OR, odds ratio; CI, confidence interval; BMI, body mass index; COPD, chronic obstructive pulmonary disease; POP-Q, Pelvic Organ Prolapse Quantification System.

## Data Availability

Anonymized data will be shared on request from any qualified investigator.
